# Supplementary material for: Why conspiracy theorists are not always paranoid: Conspiracy theories and paranoia form separate factors with distinct psychological predictors
Source: PLoS One. 2022 Apr 7;17(4):e0259053. doi: 10.1371/journal.pone.0259053 (PMC8989304; doi:10.1371/journal.pone.0259053)
Supplement: S1 Table — (DOCX) [file pone.0259053.s001.docx]

S1 Tables. Partial correlations between belief systems and psychological constructs for the three studies.

*Study 1 partial correlation coefficients between psychological predictor variables and paranoia and conspiracy mentality.*

| **Predictor Variables** | | **Paranoia** | | **Conspiracy Mentality** | |
| --- | --- | --- | --- | --- | --- |
|  |  | **r** | **Partial r** | **r** | **Partial r** |
| **Attachment** | Model of Self | -.464*** | -.455*** | -.115* | .060 |
|  | Model of Others | -.188*** | -.161** | -.110* | -.047 |
| **Self-schemas** | Positive Self | -.293*** | -.307*** | -.017 | .098* |
|  | Negative Self | .391*** | .413*** | .012 | -.147** |
|  | Positive Others | -.206*** | -.203*** | -.045 | .030 |
|  | Negative Others | .401*** | .344*** | .255*** | .131** |
| **Locus of control** | Internality | -.120* | -.118* | -.028 | .016 |
|  | Chance | .413** | .366*** | .222*** | .089 |
|  | Powerful Others | .342*** | .288*** | .223*** | .117* |
| **Loneliness** | | .518*** | .508*** | .131** | -.065 |

Note: * p < .05; ** p < .01; *** p < .001.

The partial correlations controlled for the other outcome variable (i.e., when paranoia is the predicted variable, conspiracy mentality is the control variable and vice-versa).

*Study 2 partial correlation coefficients between psychological predictor variables and paranoia and conspiracy mentality.*

| **Predictor Variables** | | **Paranoia** | | **Conspiracy Mentality** | |
| --- | --- | --- | --- | --- | --- |
|  |  | **r** | **Partial r** | **r** | **Partial r** |
| **Attachment** | Model of Self | -.434*** | -.416*** | -.148*** | .053* |
|  | Model of Others | -.164*** | -.198*** | .033 | .118*** |
| **Self-esteem** | Positive | -.242*** | -.304*** | .070** | .202*** |
|  | Negative | .729*** | .682*** | .356*** | .058* |
| **Locus of Control** | Internality | -.028 | -.065* | .070** | .091*** |
|  | Chance | .519*** | .404*** | .438*** | .273*** |
|  | Powerful Others | .542*** | .436*** | .430*** | .255*** |
| **Loneliness** | | .590*** | .539*** | .287*** | .038 |
| **Cognitive Reflection Test** | Number correct | -.095** | .008 | -.233*** | -.214*** |

Note: * p < .05; ** p < .01; *** p < .001.

The partial correlations controlled for the other outcome variable (i.e., when paranoia is the predicted variable, conspiracy mentality is the control variable and vice-versa).

*Study 3 partial correlation coefficients between psychological predictor variables and paranoia and conspiracy mentality.*

| **Predictor Variables** | | **Paranoia** | | **Conspiracy Mentality** | |
| --- | --- | --- | --- | --- | --- |
|  |  | **r** | **Partial r** | **r** | **Partial r** |
| **Attachment** | Avoidant Attachment | .039 | .034 | .020 | .000 |
|  | Attachment Anxiety | .008 | -.010 | .033 | .034 |
| **Self-esteem** | Positive | -.059 | -.096* | .048 | .090* |
|  | Negative | .468*** | .322*** | .430*** | .255*** |
| **Locus of Control** | Internality | .119** | .098* | .069 | .011 |
|  | Chance | .413*** | .243*** | .450*** | .307*** |
|  | Powerful Others | .432*** | .264*** | .455*** | .306*** |
| **Narcissism** | | .195*** | .053 | .301*** | .239*** |
| **Cognitive Reflection Test** | Number correct | -.094* | .052 | -.273*** | -.263*** |

Note: * p < .05; ** p < .01; *** p < .001.

The partial correlations controlled for the other outcome variable (i.e., when paranoia is the predicted variable, conspiracy mentality is the control variable and vice-versa).
