# Supplementary material for: Why conspiracy theorists are not always paranoid: Conspiracy theories and paranoia form separate factors with distinct psychological predictors
Source: PLoS One. 2022 Apr 7;17(4):e0259053. doi: 10.1371/journal.pone.0259053 (PMC8989304; doi:10.1371/journal.pone.0259053)
Supplement: Attachment — Submitted filename: PONE-D-21-14707 response to reviewers.docx [file pone.0259053.s005.docx]

**PONE-D-21-14707
Why Conspiracy Theorists Are Not Paranoid: Conspiracy Theories and Paranoia Form Separate Factors with Distinct Psychological Predictors**

**Rebuttal Letter**

Dear Karl,

We apologise for the further delay in revising this manuscript, which has been caused by the family circumstances of the senior author (serious illness in a family member). We thank you for your review of our submission and the two referees’ reports that you obtained. We are delighted that these are so positive and have been pleased to make the changes suggested.

Editorial advice

1. Please ensure that your manuscript meets PLOS ONE's style requirements, including those for file naming.

RESPONSE: We have done this to the best of our ability. However, we have been unable to find the journal’s guidance on file naming.

2. Please provide additional details regarding participant consent. In the ethics statement in the Methods and online submission information, please ensure that you have specified (1) whether consent was informed and (2) what type you obtained (for instance, written or verbal, and if verbal, how it was documented and witnessed). If your study included minors, state whether you obtained consent from parents or guardians. If the need for consent was waived by the ethics committee, please include this information.

RESPONSE: We have given a detailed account of our consent procedure in the participants sections of the first study, and we have included the reference number of our ethics approval, noting that the same procedure and ethics approval also applied to Studies 2 and 3.

3. Please change "female” or "male" to "woman” or "man" as appropriate, when used as a noun

RESPONSE: We have done this throughout.

4. We note that you have indicated that data from this study are available upon request. PLOS only allows data to be available upon request if there are legal or ethical restrictions on sharing data publicly. For more information on unacceptable data access restrictions

RESPONSE: We have uploaded our data files with this submission and we are happy for them to be freely accessible to other researchers.

5. Please include captions for your Supporting Information files at the end of your manuscript, and update any in-text citations to match accordingly.

RESPONSE: We have done this.

Referee’s comments

Reviewer #1: This is a very interesting paper that provide an insightful comparison between subclinical paranoia and conspiracy beliefs. I would reccomend it for acceptance.

I have just a minor observation:

1. In Study 2, you reported that the GCB has 17 items, but in the Study 3 it has 15 items. I think the latter is the correct one.

RESPONSE: This discrepancy was explained in footnotes in our originally submitted manuscript: the two extra items in study 2 originate in a study of Wood et al. (2012). We have moved this explanation into the description of the scale. A footnote in the results section, explaining that we have indeed replicated Wood et al., is now included in the main text of the results section of Study 1.

Reviewer #2: Thank you for the opportunity to review this manuscript, in which the authors further investigate the distinction between paranoia and belief in conspiracy theories. In three cross-sectional studies, they found a two-factor model of paranoid beliefs and conspiracy belief to be a better bit than a one-factor model, and that both belief systems have distinct and similar psychological factors associated with them.

Overall, I think that the manuscript reads well, the findings were very interesting, and it provides a meaningful contribute to our understanding of the relationship between paranoid beliefs and conspiracy beliefs. I have only some minor points that can be addressed:

Introduction:

There have been theoretical advancements in the conspiracy literature that were not included in the introduction. This was surprising considering the predictors variables that were included in the studies. People may be motived to believe in conspiracy theories (often unconsciously) in an attempt to satisfy/defend important psychological needs: existential (e.g., attachment insecurities), epistemic (e.g., intuitive vs analytical), social (e.g., self-esteem, narcissism; Douglas et al., 2017; see also van Prooijen, 2020). I would suggest including this framework in the conspiracy section of the introduction.

RESPONSE: We have rewritten the introductory summary of the literature on conspiracy theories, organizing it around the three factor account of Douglas et al. (2017).

Discussion:

Paranoia and belief in conspiracy theories are two distinct psychological phenomena, agreed. What does this mean for the relationship between them though? Your title states that “..conspiracy theorists are not paranoid”. Does this mean that if someone believes in conspiracy theories for paranoid reasons (i.e., that everyone is out to get them), then they are not a conspiracy theorist but just paranoid? I think a lot more can be said in the discussion to tease apart the nuance between belief in conspiracy theories and paranoia.

The discussion lacks a conclusion to tie it all in. In light Imhoff and Lamberty (2018) and the current findings, what does this mean for the relationship between paranoia and conspiracy belief going forward?

RESPONSE: We slightly have slightly changed the title so it now reads ‘Why conspiracy theorists are not always paranoid’. We have also added two substantial paragraphs to the conclusion in which we summarise our findings, address the relationship between paranoia and conspiracy theories (including the possibility that paranoia might lead to CTs) and highlight three important theoretical, practical and research implications for studies of the two belief systems going forward.
